# Heme Biosynthesis mRNA Expression Signature: Towards a Novel Prognostic Biomarker in Patients with Diffusely Infiltrating Gliomas

**DOI:** 10.3390/cancers13040662

**Published:** 2021-02-07

**Authors:** Mario Mischkulnig, Barbara Kiesel, Daniela Lötsch, Thomas Roetzer, Martin Borkovec, Lisa I. Wadiura, Karl Roessler, Shawn Hervey-Jumper, Josef M. Penninger, Mitchel S. Berger, Georg Widhalm, Friedrich Erhart

**Affiliations:** 1 Department of Neurosurgery, Medical University of Vienna, Währinger Gürtel 18–20, 1090 Vienna, Austria; mario.mischkulnig@meduniwien.ac.at (M.M.); barbara.kiesel@meduniwien.ac.at (B.K.); Daniela.Loetsch@meduniwien.ac.at (D.L.); martin.borkovec@skyforge.at (M.B.); Lisa.wadiura@meduniwien.ac.at (L.I.W.); karl.roessler@meduniwien.ac.at (K.R.); friedrich.erhart@meduniwien.ac.at (F.E.); 2 Comprehensive Cancer Center—Central Nervous System Tumours Unit, Medical University of Vienna, Währinger Gürtel 18–20, 1090 Vienna, Austria; 3 Department of Neurology, Division of Neuropathology and Neurochemistry, Medical University of Vienna, Währinger Gürtel 18–20, 1090 Vienna, Austria; Thomas.roetzer@meduniwien.ac.at; 4 Department of Neurological Surgery, University of California San Francisco, 505 Parnassus Ave, San Francisco, CA 94143, USA; Shawn.Hervey-Jumper@ucsf.edu (S.H.-J.); Mitchel.Berger@ucsf.edu (M.S.B.); 5 Institute of Molecular Biotechnology of the Austrian Academy of Sciences, Dr.-Bohr-Gasse 3, 1030 Vienna, Austria; josef.penninger@ubc.ca; 6 Department of Medical Genetics, Life Sciences Institute, University of British Columbia, C201–4500 Oak Street, Vancouver, BC V6H 3N1, Canada

**Keywords:** glioma, heme biosynthesis, TCGA, gene expression, survival

## Abstract

**Simple Summary:**

Diffusely infiltrating gliomas are frequent brain tumors with variable prognosis. In addition to the blood pigment’s role of oxygen transportation, the metabolic pathway synthesizing heme has been shown to play a role in the biochemistry of various tumors. In this study we thus investigated the impact of heme biosynthesis factors mRNA expression on the survival in glioma patients and observed a progressive decrease in survival time with increasing mRNA expression signature. This association was present for overall as well as progression-free survival and remained statistically significant after correction for established prognostic factors such as patient age and tumor grade.

**Abstract:**

Diffusely infiltrating gliomas are characterized by a variable clinical course, and thus novel prognostic biomarkers are needed. The heme biosynthesis cycle constitutes a fundamental metabolic pathway and might play a crucial role in glioma biology. The aim of this study was thus to investigate the role of the heme biosynthesis mRNA expression signature on prognosis in a large glioma patient cohort. Glioma patients with available sequencing data on heme biosynthesis expression were retrieved from The Cancer Genome Atlas (TCGA). In each patient, the heme biosynthesis mRNA expression signature was calculated and categorized into low, medium, and high expression subgroups. Differences in progression-free and overall survival between these subgroups were investigated including a multivariate analysis correcting for WHO grade, tumor subtype, and patient age and sex. In a total of 693 patients, progression-free and overall survival showed a strictly monotonical decrease with increasing mRNA expression signature subgroups. In detail, median overall survival was 134.2 months in the low, 79.9 months in the intermediate, and 16.5 months in the high mRNA expression signature subgroups, respectively. The impact of mRNA expression signature on progression-free and overall survival was independent of the other analyzed prognostic factors. Our data indicate that the heme biosynthesis mRNA expression signature might serve as an additional novel prognostic marker in patients with diffusely infiltrating gliomas to optimize postoperative management.

## 1. Introduction

Diffusely infiltrating gliomas constitute the most frequent malignant primary tumor entity of the central nervous system (CNS) [1,2]. Routinely, histopathological grading of these tumors is performed according to the World Health Organization (WHO) classification into three separate tumor grades (WHO grades II–IV) [3,4]. In the last years, further characterization of gliomas with assistance of molecular markers has been increasingly conducted [3]. Aside from these molecular markers, the WHO tumor grades are still important parameters for estimation of the patient prognosis with WHO grade IV gliomas constituting the most aggressive tumor with a median overall survival of less than two years [5,6]. In contrast, lower-grade gliomas show distinctly more favorable median overall survival times of 6.5 years (WHO grade II) and 3.1 years (WHO grade III), respectively [7]. In addition to the WHO tumor grades, additional prognostic biomarkers such as tumor subtypes including histopathological subtypes (astrocytic vs. oligodendroglial tumors) as well as Verhaak tumor subtypes (classical, neural, proneural, and mesenchymal), and patients’ age and sex are of clinical relevance in gliomas [1,8,9,10]. However, further clinically reliable prognostic biomarkers are urgently needed to optimize the postoperative management in glioma patients.

The heme biosynthesis cycle represents a fundamental pathway in human cells and is substantially involved in multiple cellular functions [11,12]. In a recent in silico investigation of heme biosynthesis pathway mRNA expression between WHO grade II and IV gliomas, we found differences in the mRNA expression in 8 of 11 analyzed enzymes/transporters [13]. In this sense, different gene expression between WHO grade II and grade IV gliomas was found in the Solute Carrier Family 15 Member 2 (SLC15A2), ALA-Dehydratase (ALAD), Hydroxymethylbilane Synthase (HMBS), Uroporphyrinogen-III-Synthase (UROS), Uroporphyrinogen-Decarboxylase (UROD), ATP-binding Transporter Cassette Subfamily B Member 6 (ABCB6), Protoporphyrinogen-Oxidase (PPOX), and Ferrochelatase (FECH) [13]. Based on these data, we introduced in our previous study the “mRNA expression signature” characterizing the heme biosynthesis expression in diffusely infiltrating gliomas [13]. Since we observed distinct heme biosynthesis mRNA expression differences between WHO grade II and grade IV gliomas in our previous study [13], the logical next step was to assess a potential impact of the heme biosynthesis mRNA expression signature on the prognosis in patients suffering from diffusely infiltrating gliomas.

The aim of the present study was thus to investigate the patient prognosis in diffusely infiltrating gliomas in dependence of the heme biosynthesis pathway. To that end, we evaluated the recently established heme biosynthesis mRNA expression signature and correlated these data with patient survival. In order to achieve a substantial cohort of patients with WHO grades II–IV gliomas, we relied on the tumor tissue database provided by “The Cancer Genome Atlas” (TCGA) framework, where comprehensive mRNA expression data are available [14,15,16].

## 2. Materials and Methods

In the present study, we analyzed data from the TCGA (PANCAN and Glioblastoma datasets) [17,18,19] of the National Cancer Institute and Human Genome Research Institute. Data retrieval was performed using the “Xena tool” developed by the Genomics Institute at the University of California Santa Cruz, USA [20]. The PANCAN dataset was filtered for histopathologically verified diffusely infiltrating gliomas (glioblastoma, oligodendroglioma, astrocytoma, or oligoastrocytoma) and distinct WHO tumor grades II, III, or IV. We only included patients with available mRNA sequencing data on heme biosynthesis factors in the current study. The analysis of specific tumor characteristics including the heme biosynthesis factor expression on patient survival was approved by the Medical University of Vienna’s ethics committee (EK 419/2008—amendment).

### 2.1. Analysis of the Heme Biosynthesis mRNA Expression Signature

Based on the TCGA sequencing data, we analyzed the mRNA expression in a total of 11 relevant heme biosynthesis factors including seven enzymes (ALAD, HMBS, UROS, UROD, CPOX, PPOX, and FECH) and four transporters (SLC15A1, SLC15A2, ABCB6, and ABCG2). As integrative parameter describing the complex relation of the expression of these heme biosynthesis factors in diffusely infiltrating gliomas, we utilized the recently established heme biosynthesis “mRNA expression signature” that constitutes the linear formula most accurately describing differential mRNA expression of WHO grade II and IV gliomas [13]. In detail, the mRNA expression signature is based on the following formula [13] using log-transformed normalized mRNA expression data in log2 (norm_value+1):

mRNA expression signature = 0.107*Log2(norm_value + 1)SLC15A1 − 1.255*Log2(norm_value + 1)SLC15A2 − 2.223* Log2(norm_value + 1)ALAD +2.634* Log2(norm_value + 1)HMBS-2.928* Log2(norm_value + 1)UROS + 2.988* Log2(norm_value + 1)UROD-1.428* Log2(norm_value + 1)ABCB6 + 0.659* Log2(norm_value + 1)CPOX − 2.863* Log2(norm_value + 1)PPOX-0.476* Log2(norm_value + 1)ABCG2 + 2.488* Log2(norm_value + 1)FECH

### 2.2. Analysis of Survival Data

In each patient, we investigated the progression-free survival as well as overall survival to evaluate if the heme biosynthesis mRNA expression signature has a prognostic impact in glioma patients. For survival analyses, patients were categorized into equally sized “low” (<33.3rd percentile), “medium” (33.3rd-66.6th percentile) and “high” (>66.6th percentile) expression subgroups according to the mRNA expression signature values. Further, to rule out artifacts due to these arbitrarily set subgroups, results of the main analysis were confirmed using the mRNA expression signature as a continuous variable. For correlation of survival data with other prognostic factors in the multivariate analysis, we retrieved data on the WHO grade, histopathological subtype (astrocytoma, oligodendroglioma, and oligoastrocytoma), Verhaak tumor subtype (classical, parenchymal, proneural, and neural) [21], and patient age and sex from the TCGA datasets.

### 2.3. Statistical Analysis

Statistical investigations were conducted using SPSS statistical software (Version 26.0, SPSS Inc.). Descriptive statistical measurements included patient age, sex distribution, WHO tumor grades and tumor subtypes. Survival analyses were subsequently performed for progression-free survival as well as overall survival with respect to the above described mRNA expression signature subgroups. These respective calculations were conducted for the entire cohort as well as for each WHO tumor grade separately using Kaplan–Meier analyses with log-rank testing. Additionally, a Cox regression model including multiple possible confounders/prognostic factors such as WHO tumor grade, histopathological subtypes in WHO grade II/III gliomas (astrocytoma, oligodendroglioma, and oligoastrocytoma), Verhaak tumor subtypes in WHO grade IV gliomas, patient age and sex was calculated in the multivariate analysis—considering all potential confounders equally and consistently. All cases fulfilling the formal criteria of the Cox regression model (data for all covariates available, survival not censored before the earliest event in the respective subgroup) were included in the multivariate analysis. In order to illustrate our results, Kaplan–Meier curves were created for survival in the entire cohort and for subgroups stratified by the WHO tumor grade as well as estimated by the Cox regression model. The threshold of statistical significance was set at the commonly applied value of *p* < 0.05 and all *p*-values of the main study investigating patient survival were adjusted for multiple testing using Bonferroni correction. In addition to the main study investigating the survival impact of mRNA expression signature on survival, an exploratory data analysis of mRNA expression signature distribution in the investigated possible confounders was conducted.

## 3. Results

In the present study, we identified a total of 693 patients with diffusely infiltrating gliomas (WHO grades II–IV) and available data on heme biosynthesis mRNA expression and survival. The median patient age was 46 years (range 14–89 years) and 397 (57.3%) patients were male and 296 (42.7%) female. With regard to the WHO tumor grade, the study cohort consisted of 257 WHO grade II, 270 WHO grade III, and 166 WHO grade IV gliomas. With reference to the tumor subtype, astrocytomas and oligodendrogliomas accounted for 197 (37.4%) tumors each and the remaining 133 (25.2%) cases were classified as oligoastrocytomas within the subgroup of WHO grade II and III gliomas. The Verhaak subtype was available for 163 (98.2%) WHO grade IV gliomas and the mesenchymal subtype was most commonly observed (55 patients, 33.1%) compared to the classical (42 patients, 25.3%), proneural (38 patients, 22.9%), and neural (28 patients, 16.9%) subtypes. Detailed patient characteristics are provided in Table 1.

### 3.1. Heme Biosynthesis mRNA Expression Signature and Subgroups

In the entire study cohort of 693 gliomas, the median heme biosynthesis mRNA expression signature was −24.62 (range: −32.69 to −7.82) with 33rd percentile at −26.7237 and 66th percentile at −22.2880, respectively. Therefore, the mRNA expression subgroups for subsequent survival analyses were set accordingly as low (mRNA expression signature: ≤−26.7237), intermediate (mRNA expression signature between −26.7237 and −22.2880) and high (mRNA expression signature: >−22.2880). 

### 3.2. Heme Biosynthesis mRNA Expression Signature and Progression-Free Survival

**Figure 1:**
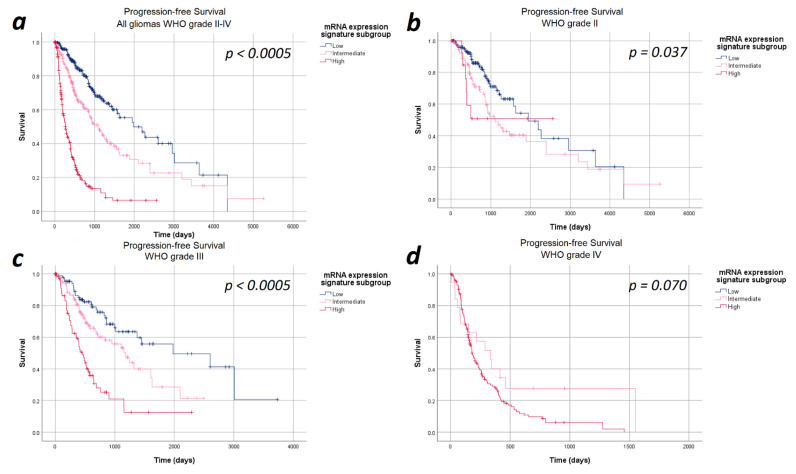
Kaplan–Meier curves of progression-free survival stratified by heme biosynthesis mRNA expression signature subgroups. (**a**) All gliomas WHO grade II–IV: The progression-free survival differed significantly between the mRNA expression signature subgroups in the entire study cohort. (**b**) In WHO grade II gliomas no significant differences in progression-free survival between the mRNA expression signature subgroups was present. (**c**) In WHO grade III, a significantly poorer survival was observed with increasing mRNA expression signature subgroups. (**d**) WHO grade IV gliomas: We did not find a statistically significant difference in the progression-free survival between the mRNA expression signature subgroups in WHO grade IV gliomas.

The median progression-free survival in the entire cohort of 693 patients was 27.6 months (95%CI: 23.7–31.9 months). In the entire study cohort, progression-free survival differed significantly between the heme biosynthesis mRNA expression signature subgroups with a strictly monotonical decrease in the median progression-free survival with increasing mRNA expression signature (*p* < 0.0005). In detail, the median progression-free survival was 65.0 months within patients in the low, 36.3 months in the intermediate and 8.6 months in the high mRNA expression signature subgroups. After stratifying the analysis for the WHO tumor grades, the strictly monotonical decrease of the progression-free survival with increasing mRNA expression signature subgroups remained present within all WHO tumor grades. However, the decreases in progression-free survival with increasing mRNA expression signature were only statistically significant within WHO grades III (*p* < 0.0005) gliomas, but not within WHO grade II (*p* = 0.370) and IV (*p* = 0.700) gliomas. Kaplan–Meier curves of the progression-free survival are provided in Figure 1 and detailed data are given in Table 2. 

### 3.3. Heme Biosynthesis mRNA Expression Signature and Overall Survival

**Figure 2:**
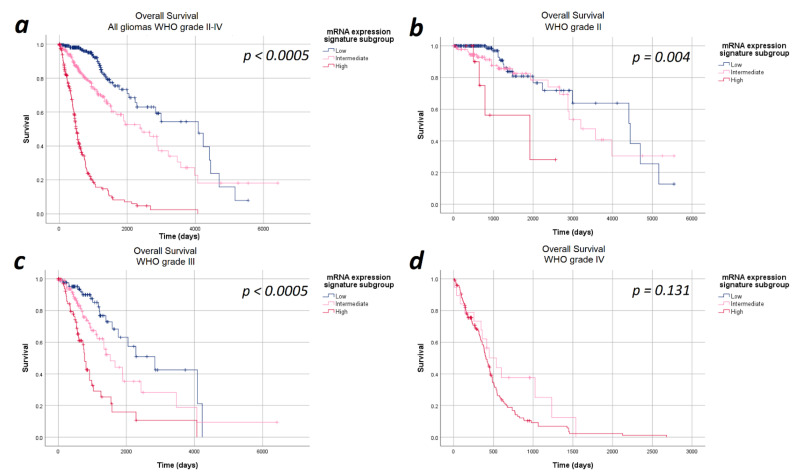
Kaplan–Meier curves of overall survival stratified by heme biosynthesis mRNA expression signature subgroups. (**a**) All gliomas WHO grades II–IV: The overall survival differed significantly between the mRNA expression signature subgroups in the entire study cohort. (**b**) In WHO grade II and (**c**) WHO grade III gliomas significant differences in the overall survival between the mRNA expression signature subgroups were found. (**d**) WHO grade IV gliomas: No statistically significant difference in the overall survival between the mRNA expression signature subgroups was observed in WHO grade IV gliomas.

The median overall survival in the entire cohort of 693 patients was 48.7 months (95%CI: 38.2–59.1 months). Overall, the median overall survival showed a statistically significant difference in the heme biosynthesis mRNA expression signature subgroups with a strictly monotonical decrease in the median overall survival with increasing mRNA expression signature (*p* < 0.0005). The median overall survival was 134.2 months in the low, 79.9 months in the intermediate and 16.6 months in the high mRNA expression signature subgroups. Stratification of the survival curves according to the WHO tumor grade demonstrated a survival impact of the heme biosynthesis mRNA expression signature independent of the WHO tumor grade with a monotonical decrease in median overall survival. In analogy to the findings with regard to progression-free survival, the observed overall survival differences were statistically significant within WHO grades II (*p* = 0.040) and III gliomas (*p* < 0.0005), but not within WHO grade IV (*p* = 1.000) gliomas. The Kaplan–Meier curves of overall survival are shown in Figure 2 and detailed data are given in Table 2. 

### 3.4. Multivariate Analysis

**Figure 3:**
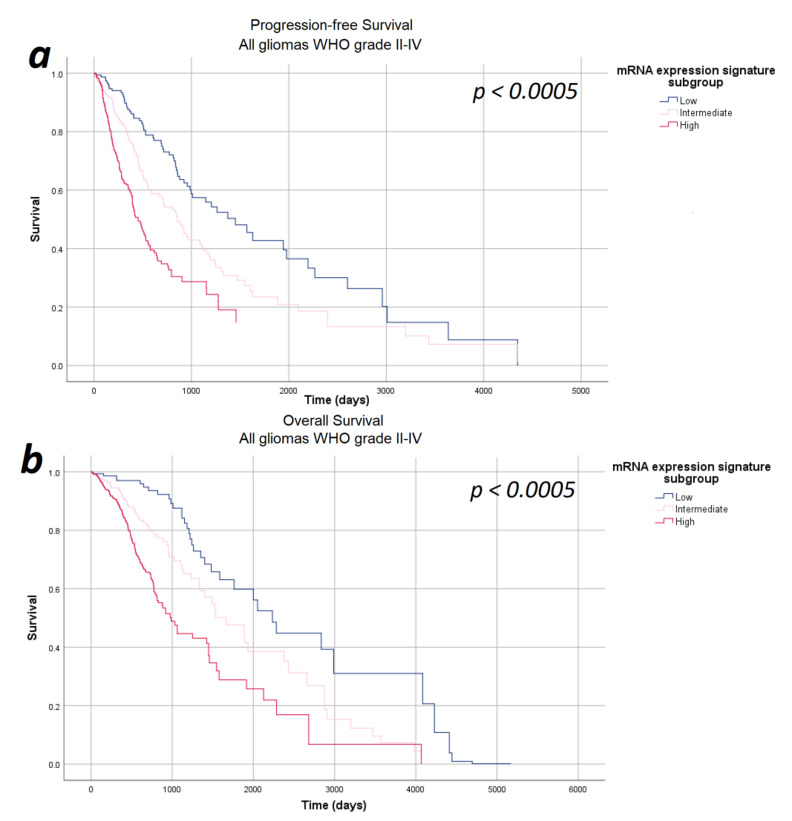
Survival-curves for progression-free survival and overall survival after correction for WHO tumor grade, tumor subtype, and patient age and sex as estimated by the multivariate analysis. (**a**) Progression-free survival and (**b**) overall survival: The mRNA expression signature significantly correlated with the progression-free survival as well as the overall survival after correcting for the WHO tumor grade, tumor subtype, and patient age and sex.

In the next step, we investigated the impact of the mRNA expression signature on survival in a multivariate analysis including relevant prognostic factors, such as WHO tumor grade, tumor subtype, and patients’ age and sex—thus ensuring that all potential confounders are considered equally and consistently in a single, integrated model. The multivariate analyses were based on 671 patients (progression-free survival) and 673 patients (overall survival) fulfilling the requirements of the Cox regression model described in the Methods section. According to our multivariate analysis, the mRNA expression signature significantly correlated with both progression-free survival as well as overall survival after correcting for the WHO tumor grade, tumor subtype, age, and sex (*p* < 0.0005). Survival curves for the Cox model for progression-free and overall survival are given in Figure 3. In order to verify that our results demonstrate a genuine prognostic impact rather than an artifact due to the arbitrarily defined subgroups, Cox regression results were verified using the continuous mRNA expression signature variable and the *p*-values of *p* < 0.0005 were confirmed.

### 3.5. Exploratory Data Analysis of mRNA Signature Distribution in Relevant Prognostic Factors

**Figure 4:**
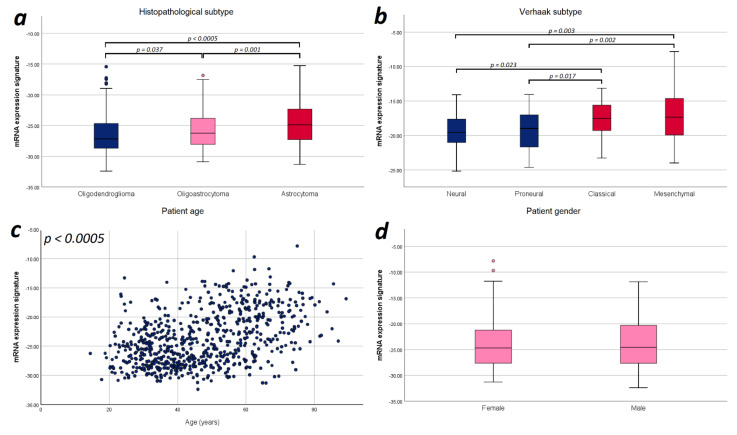
Illustration of the distribution of mRNA expression signature according to relevant prognostic factors. (**a**) Histopathological subtype: Significant differences in the mRNA expression signature were found between astrocytomas, oligoastrocytomas and oligodendrogliomas. (**b**) Verhaak tumor subtype: The mesenchymal and classical Verhaak tumor subtypes showed a significantly higher mRNA expression signature compared to neural and proneural subtypes. (**c**) Patient age: The mRNA expression signature significantly correlated with the patient age. (**d**) Patient sex: No statistically significant difference in the mRNA expression signature was observed between female and male patients. Only *p*-values with significant differences were included in this figure.

Finally, we explored potential correlations of the mRNA expression signature with the prognostic factors applied in the multivariate analysis for an improved understanding of glioma biology. Since we observed a strictly monotonical association of the mRNA expression signature with the WHO tumor grade in our previous study [21], we performed further correlations focusing on tumor subtype, and patient age and sex. In WHO grade III gliomas, we found a significant difference in the mRNA expression signature between astrocytomas, oligoastrocytomas and oligodendrogliomas (−23.74 vs. −24.72 vs. −25.77; *p* < 0.0005). For WHO grade II gliomas, we did not observe such difference (−26.41 vs. −26.57 vs. −27.03; *p* = 0.137). With respect to WHO grade IV gliomas, we found that mesenchymal and classical Verhaak tumor subtypes showed a significantly higher mRNA expression signature compared to neural (mesenchymal vs. neural: *p* = 0.003; classical vs. neural: *p* = 0.023) and proneural subtypes (mesenchymal vs. proneural: *p* = 0.002; classical vs. proneural: *p* = 0.017). Furthermore, the mRNA expression signature significantly correlated with the patient age (*r* = 0.431; *p* < 0.0005), but no difference between female and male patients was observed (*p* = 0.718). Illustration of these correlations are provided in Figure 4. 

## 4. Discussion

The heme biosynthesis cycle constitutes a fundamental metabolic pathway in human cells generating heme which is substantially involved in multiple cellular function [11,22]. In addition to its well-known role in the oxygen binding of hemoglobin and myoglobin, heme is also necessary for the function of the aerobic energy metabolism as part of the Cytochrome-c-oxidase of the respiratory chain [23,24]. Alterations in the energy metabolism, specifically a metabolic shift from mitochondrial oxidative phosphorylation to aerobic glycolysis, constitute a characteristic hallmark of glioma biology [25]. In this sense, we recently observed significant differences in the mRNA expression in 8 of 11 analyzed genes (HMBS, UROD, FECH, PPOX, SLC15A2, ALAD, UROS, and ABCB6) involved in the heme biosynthesis between WHO grade II and IV gliomas [13]. According to these data, we introduced the “mRNA expression signature” characterizing the heme biosynthesis expression in diffusely infiltrating gliomas [13]. Since we found distinct differences in heme biosynthesis mRNA expression between different WHO tumor grades and normal brain tissue with continuously increasing values with higher tissue anaplasia [13] and a prognostic impact of heme biosynthesis for several malignancies including gliomas was suggested in a recent exploratory study [26], we thus hypothesized that the heme biosynthesis mRNA expression signature might constitute an independent prognostic marker in patients with diffusely infiltrating gliomas.

### 4.1. Present Study

We therefore designed the present study to investigate the prognosis of patients suffering from diffusely infiltrating gliomas (WHO grades II–IV) in dependence of the heme biosynthesis mRNA expression signature in a large TCGA patient cohort. According to our data, we observed a significant correlation of the heme biosynthesis mRNA expression signature with both progression-free survival as well as overall survival. Most notably, the observed median survival times showed a strictly monotonical association with the mRNA expression signature, even within different WHO tumor grades. The findings of this first systematical analysis focusing on gliomas thus demonstrate the impact of the heme biosynthesis mRNA expression signature on patient survival. Our current data from this large TCGA glioma patient cohort are in accordance with the initial report suggesting a potential prognostic role of the heme biosynthesis pathway in different tumor entities [26]. Therefore, the heme biosynthesis mRNA expression signature might serve as a novel prognostic marker to improve the postoperative management of glioma patients in future. 

### 4.2. Heme Biosynthesis mRNA Expression Signature as Independent Prognostic Factor

A number of various prognostic factors have been identified in glioma patients such as patient age, sex, Karnofsky performance score (KPS), extent of resection, WHO tumor grade, and tumor subtypes [9,27]. In order to determine the role of the heme biosynthesis regulation as an independent prognostic factor, we thus conducted a multivariate analysis correcting for possible factors with plausible confounding effects on tumor biology and available data in the TCGA PANCAN dataset. KPS and extent of resection were not considered given that they are not directly related to tumor biology. After correction for patient age, sex, WHO tumor grade, and tumor subtypes in our multivariate analysis, the impact of the heme biosynthesis mRNA expression signature on glioma patient prognosis remained highly significant. Therefore, heme biosynthesis mRNA expression signature represents an independent prognostic marker in patients with diffusely infiltration gliomas.

### 4.3. Heme Biosynthesis mRNA Expression Signature and Aggressive Tumor Biology

In order to improve the understanding of tumor biology in diffusely infiltrating gliomas, we also investigated the relation between the mRNA expression signature and tumor subtypes (astrocytoma/oligoastrocytoma/oligodendroglioma for WHO grade II/III gliomas and Verhaak classification for WHO grade IV gliomas). According to our data, we found a significant difference in the mRNA expression signature between specific histopathological subtypes in WHO grade III gliomas with highest levels in astrocytomas as well as increased mRNA expression signature in mesenchymal/classical compared to neural/proneural Verhaak tumor subtypes. While the exact underlying mechanisms leading to higher mRNA expression signature in certain glioma subtypes are not entirely clear, the presence of elevated values in more aggressive histopathological subtypes as well as more aggressive Verhaak tumor subtypes seems to be another indicator that the heme biosynthesis pathways plays a relevant role in glioma aggressiveness and should thus be further investigated. 

### 4.4. Heme Biosynthesis mRNA Expression and 5-ALA Fluorescence

Nowadays, 5-aminolevulinic acid (5-ALA) is widely applied for intraoperative visualization of high-grade gliomas (WHO grades III and IV) with assistance of visible fluorescence to improve the extent of tumor resection [28,29]. Routinely, patients receive 5-ALA perorally approximately three hours prior to surgery and specifically modified microscopes with violet-blue excitation light are used for fluorescence visualization during surgery [30,31]. The intraoperative fluorescence effect is dependent on the conversion of 5-ALA to fluorescent Protoporphyrin IX and is therefore likely facilitated by enzymes of the heme biosynthesis pathway and may also be impacted by the eventual enzymatic breakdown of heme [32,33]. In addition to the locally increased concentration of heme biosynthesis metabolites in glioma tissue, higher urine and plasma concentrations of PpIX have been identified in patients with fluorescent gliomas compared to those that showed no or only minimal fluorescence [34,35]. Since high-grade gliomas (WHO grades III and IV) usually demonstrate visible 5-ALA fluorescence and such fluorescence is frequently absent in low-grade gliomas (WHO grade II) as well as 5-ALA is metabolized via the heme biosynthesis pathway, we assume that 5-ALA fluorescence might be a reliable intraoperative indicator for the heme biosynthesis mRNA signature [28,36,37,38]. Therefore, the 5-ALA fluorescence might allow the neurosurgeon an immediate intraoperative estimation of the heme biosynthesis mRNA signature and thus patient prognosis. Future studies should thus focus on investigating the relation between 5-ALA fluorescence and heme biosynthesis mRNA expression signature in glioma patients.

### 4.5. Clinical Relevance

First of all, the heme biosynthesis mRNA expression signature might constitute a valuable addition to the currently available prognostic markers in glioma patients. Furthermore, the heme biosynthesis mRNA expression signature might improve the postoperative patient management. The early initiation of postoperative treatment in low-grade gliomas with radio- and/or chemotherapy still remains a matter of discussion in the routine clinical practice. Since we found a significantly lower median overall survival in low-grade glioma patients with high (63 months) vs. low/intermediate (131 months) heme biosynthesis mRNA expression signature, this important information might support the selection of low-grade glioma patients requiring early initiation of postoperative adjuvant treatment. Moreover, the underlying alterations in the heme biosynthesis pathway might represent potential targets for future therapeutical approaches, and thus should be investigated in future studies. 

### 4.6. Limitations

The following limitations should be considered: (1) First of all, the current study is based on a single cohort. Thus, our findings should be further validated by future experiments in an independent cohort. (2) Furthermore, our current analysis is limited by its retrospective study design and comprehensive inclusion of glioma patients rather than selection of a highly homogeneous cohort by factors such as comorbidities. Therefore, future prospective studies should be performed to confirm our present data on the prognostic impact of heme biosynthesis regulation in glioma patients and detect even minor survival differences. However, we are confident that our chosen study design is capable of demonstrating the presence of a fundamental prognostic impact as it is well-powered, and the results were verified by complementary statistical methods. (3) Stratification of mRNA expression signature into three subgroups was set arbitrarily and definition of fewer or more subgroups may have resulted in slightly different results. However, the Cox regression models for PFS as well as OS were repeated with the continuous mRNA signature variable and the highly significant correlations with patient survival were verified this way. (4) Finally, we did not correlate the heme biosynthesis mRNA expression signature with molecular markers in the present study. Nowadays, molecular markers are increasingly applied for further characterization of gliomas aside from the WHO tumor grades.^3^ Consequently, we plan to correlate the heme biosynthesis mRNA expression signature with specific molecular markers such isocitrate dehydrogenase (IDH) mutations, 1p/19q co-deletion and p53 mutation in the next step.

## 5. Conclusions

In the present study, we investigated the patient prognosis in WHO grade II, III, and IV gliomas in dependence of the recently established mRNA expression signature based on the heme biosynthesis pathway in a large cohort of TCGA glioma patients. Most importantly, we found a significant correlation of the heme biosynthesis mRNA expression signature with both progression-free survival as well as overall survival in diffusely infiltrating gliomas. The prognostic impact of the heme biosynthesis mRNA expression signature remained highly significant even after correction for other known prognostic factors such as WHO tumor grade or histopathological/Verhaak tumor subtypes. Thus, the heme biosynthesis mRNA expression signature might serve as an additional novel prognostic marker to optimize postoperative glioma patient management and its underlying alterations might lead to the development of future therapeutical approaches. 

## Figures and Tables

**Table 1:** Patient characteristics. Distribution of patient age, sex, histopathological, and Verhaak tumor subtypes and mRNA expression signature subgroups are shown for the entire cohort as well as distinct WHO glioma grades.

	Patient Characteristics	Overall		WHO Grade II		WHO Grade III		WHO Grade IV	
		*n*	(%)	*n*	(%)	*n*	(%)	*n*	(%)
Number of patients		693	(100)	257	(100)	270	(100)	166	(100)
Age									
	median (range)	47 (14–89)		38 (14–87)		45 (22–76)		61 (21–89)	
Sex									
	female	396	(57.3)	119	(46.3)	118	(43.7)	59	(35.5)
	male	297	(42.7)	138	(53.7)	152	(56.3)	107	(64.5)
Histopathological subtype									
	astrocytoma	197	(28.4)	65	(25.3)	132	(48.9)	-	-
	oligodendroglioma	197	(28.4)	115	(44.7)	82	(30.4)	-	-
	oligoastrocytoma	133	(19.2)	77	(30.0)	56	(20.7)	-	-
	missing data	166	(24.0)	-	-			166	(100)
Verhaak tumor subtype									
	neural	28	(4.0)	-	-	-	-	28	(16.9)
	proneural	38	(5.5)	-	-	-	-	38	(22.9)
	classical	42	(6.1)	-	-	-	-	42	(25.3)
	mesenchymal	55	(7.9)	-	-	-	-	55	(33.1)
	missing data	530	(76.5)	257	(100)	270	(100)	3	(1.8)
mRNA expression signature subgroup									
	low	231	(33.3)	143	(55.7)	88	(32.6)	-	-
	intermediate	230	(33.2)	100	(38.9)	111	(41.1)	19	(11.4)
	high	232	(33.5)	14	(5.4)	71	(26.3)	147	(88.6)

**Table 2:** Survival data. The exact case numbers and survival times underlying the Kaplan–Meier curves shown in Figure 1 and Figure 2 are provided for progression-free and overall survival.

Progression-Free Survival								
WHO Grade	mRNA Subgroup	Overall	Progression		Median			
					Estimate	Std. Error	95% CI†	
		*n*	*n*	(%)			lower	upper
II	Low	143	36	25.2	63.8	12.0	40.4	87.3
	Intermediate	100	47	47.0	36.6	6.0	24.8	48.4
	High	14	6	42.9	-	-	-	-
	Overall	257	89	34.6	51.5	9.2	33.5	69.6
III	Low	88	29	33.0	65.0	23.2	19.4	110.5
	Intermediate	111	42	37.8	38.9	5.6	28.0	49.8
	High	71	46	64.8	14.9	1.6	11.7	18.1
	Overall	270	117	43.3	33.2	3.9	25.5	40.9
IV	Low	0	-	-	-	-	-	-
	Intermediate	19	14	73.7	11.0	2.9	5.4	16.6
	High	147	119	81.0	6.0	0.6	4.8	7.1
	Overall	166	133	80.1	6.3	0.6	5.1	7.5
Overall		693	339	48.9	27.6	2.2	23.3	31.9
**Overall Survival**								
WHO Grade	mRNA subgroup	Overall	Deaths		Median			
					Estimate	Std. Error	95% CI†	
		*n*	*n*	(%)			lower	upper
II	Low	143	16	11.2	146.0	28.7	89.8	202.3
	Intermediate	100	20	20.0	105.1	13.6	78.5	131.7
	High	14	4	28.6	62.9	29.3	5.4	120.4
	Overall	257	40	15.6	117.3	21.1	76.0	158.6
III	Low	88	20	22.7	93.1	20.3	53.3	132.9
	Intermediate	111	34	30.6	50.1	7.5	35.3	64.9
	High	71	39	54.9	25.5	1.3	22.9	28.0
	Overall	270	93	34.4	50.8	4.5	42.1	59.6
IV	Low	0	-	-	-	-	-	-
	Intermediate	19	14	73.7	17.6	3.9	10.1	25.2
	High	147	119	81.0	13.3	0.8	11.7	14.9
	Overall	166	133	80.1	13.8	0.8	12.1	15.4
Overall		693	266	38.4	48.7	5.3	38.2	59.1

† CI: Confidence interval, Std ErroR: Standard Error.

## Data Availability

The data that support the findings of this study are openly available in the TCGA database accessible via the Xena Browser at https://xena.ucsc.edu/within (accessed on 2 July 2020) the TCGA PANCAN and GBMLGG datasets.
